# Demineralization of teeth in mouth-breathing patients undergoing maxillary expansion

**DOI:** 10.1590/S1808-86942010000600007

**Published:** 2015-10-19

**Authors:** Silvia Fuerte Bakor, Julio César Motta Pereira, Silvana Frascino, Thereza Christinna Cellos Gonçalves Pinheiro Ladalardo, Shirley Shizue Nagata Pignatari, Luc Louis Maurice Weckx

**Affiliations:** 1 Doctoral degree in Sciences, UNIFESP. Professor of the Specialization Orthodontics Course, FAPI; 2 Doctoral degree in Sciences, UNIFESP-EPM. Adjunct professor of the Dentistry Course, Feira de Santana State University; 3 Specialist in Pediatric Dentistry, volunteer dentist at the Mouth-Breather Unit (Centro do Respirador Bucal), Pediatric ENT Department, UNIFESP; 4 Doctoral degree in Sciences, UNIFESP. Professor at the APCD; 5 Doctoral degree, head of the Pediatric ENT Discipline, UNIFESP-EPM; 6 Full professor in the Pediatric ENT Discipline, Otorhinolaryngology and Head & Neck Department, UNIFESP-EPM, Coordinator of the graduate program of the Otorhinolaryngology and Head & Neck Department, UNIFESP-EPM

**Keywords:** tooth demineralization, lasers, mouth breathing, palatal expansion technique

## Abstract

Mouth breathing may cause deformities on the dental arch and be a risk factor for caries and periodontal disease; fixed orthodontic appliances compound the problem.

**Aim:**

to evaluate mineralization of tooth enamel and the oral cariogenic microbiota of mouth breathers that are using maxillary expanders.

**Material and method:**

a prospective study of 20 mouth-breathing patients with maxillary atresia, aged from 09 to 13 years. Enamel mineralization was measured using a fluorescence technique, before installing the expander and after its removal. The cariogenic microbiota was evaluated by the No Caries®. The t test (p<0.05) was applied for the statistical analysis, and the oral microbiota was analyzed by incidence.

**Results:**

there was a statistically significant difference in the enamel mineralization level after maxillary expansion; the mean value was 3.08. The colorimetric test showed that the caries development potential was reduced in 45%, increased in 15%, and unaltered in 40% after maxillary expander use.

**Conclusion:**

there was a statistically significant difference in enamel mineralization after maxillary expansion; this difference was within the clinically normal range; the cariogenic potential increased in a small number of patients during orthodontic treatment.

## INTRODUCTION

The mouth-breather syndrome is frequent in childhood, and may be characterized by fatigue, daytime drowsiness, adynamia, poor appetite, nighttime enuresis, and impaired learning and attention. These symptoms result from upper airway obstruction,^1^ which may often result in dentofacial deformities.^2^

Mouth breathing is also a risk factor for dental caries and periodontal disease.^3^, ^4^ The precise mechanisms are not fully understood, but probable causes are gingival surface dehydration, decreased epithelial resistance to bacterial plaques, and lack of salivary auto-cleaning.^5^ Orthodontic appliances increase the risk of dental caries and gingivitis.^6^

Although some authors have recognized that oral health has improved in the past few decades,^7^, ^8^, ^9^ dental caries remain a serious public health issue not only in Brazil, but in most of the world.^10^, ^11^ Studies have assessed dental caries calibration and enamel decalcification^12^, ^13^, ^14^ based on World Health Organization criteria, such as examining the lesions at initial phases to yield reliable and accurate results for reporting purposes. There are few studies evaluating the potential for caries in high-risk subjects - such as mouth breathers - using fixed orthodontic/orthopedic appliances. These variables may alter the occurrence and severity of caries and periodontal disease. We underline the importance of a multidisciplinary approach for treating these patients effectively.

Thus, the purpose of this study was to conduct a clinical experimental prospective cross-sectional study to assess quantitatively the degree of mineralization of dental enamel and the cariogenic microbiota of mouth breathers that were using fixed maxillary dysjunction appliances.

## MATERIAL AND METHODS

The institutional review board of the institution in which the study was carried out evaluated and approved the study project.

1. Sample: there were 20 patients of both sexes, aged from 9 to 13 years, mouth breathers with at least one of the following respiratory conditions: allergic rhinitis, adenoid hyperplasia, palatine tonsil hyperplasia, or nasal septal deviation. All patients had maxillary atresia for which orthodontic/orthopedic maxillary dysjunction was indicated. Patients who had previously been treated orthodontically or orthopedically were excluded. During this study, sample patients were monitored otorhinolaryngologically at the ENT Department of the institution, although still mouth breathing throughout the study period. Clinical dental treatment was also given during orthodontic therapy to improve and adapt mouth conditions in this group of patients.
2. Measuring the degree of mineralization of dental tissues: a Diagnodent® (Kavo, Germany) 1 mW emitting power / 655 nm wavelength / 0 to 99 fluorescence intensity laser diode device was used. The caries detection method with the 655 nm laser diode fluorescence technique measures the amount of demineralization of dental tissues (enamel and dentin); in such cases, organic matter increases and mineral content decreases in these tissues - the fluorophores in organic portions fluoresce. We measured the vestibular surfaces, neck, middle and incisive portions of upper central incisives, and the neck, middle and occlusal portions of the upper first molars before placing and after removing a maxillary dysjunction appliance. The clinical parameters for the DIAGNOdent® are provided as a reference, according to the degree of mineralization, with sensitivity and specificity values.
3. Qualitative bacteriological tests of dental caries: samples of saliva were collected at two times; before installing the maxillary dysjunction appliance, and just before its removal. Patients were asked to eat nothing for two hours, not to perform oral hygiene, and then to produce saliva (non-stimulated) and spit into a sterile universal sample collector. The first pipette was inoculated with 200 μml of saliva, which was dissolved in No Caries® 1. The second pipette was inoculated with 200 μml of saliva, which was dissolved in No Caries® 2. The test tubes were shaken for 15 seconds and placed in an incubator at 37° during 60 minutes. The results were interpreted using a colorimetric reaction scale. Yellow means a NEGATIVE potential for caries, orange means positive (+) that the manufacturer considers as carrying an AGGRESSIVE potential for caries, and pink means positive (++) that the manufacturer considers as VERY AGGRESSIVE potential for caries.
4. Maxillary dysjunction appliance: a Morelli Hyrax-type maxillary dysjunction appliance with an 0.8 mm expanding screw and cemented bands with glass ionomer cement (Sci Farm, USA) with gradual fluoride release was used.
5. Statistics: The numerical values - degree of mineralization - were compared by applying the “t” test for paired samples at a 5% significance level. The cariogenic potential was distributed by incidence.

## RESULTS

**Chart 1:**
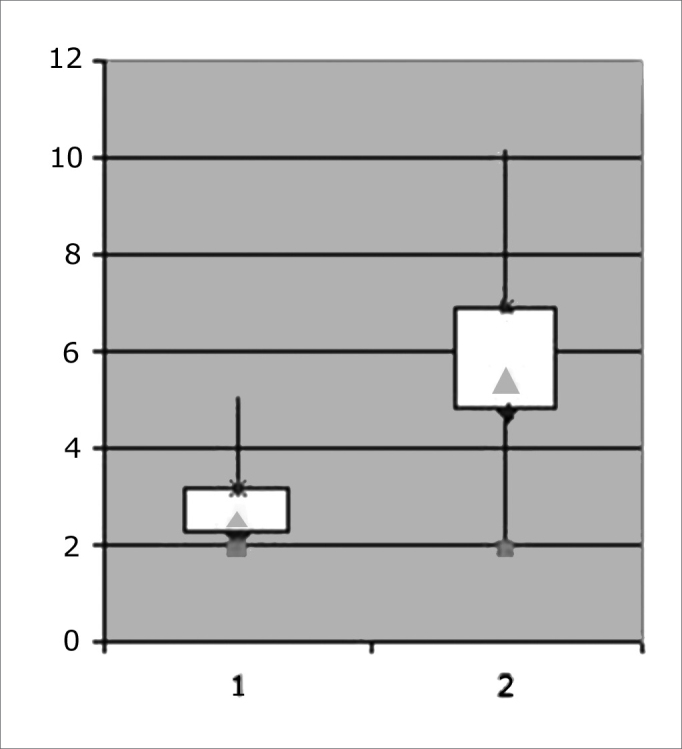
Behavior of the sample before and after maxillary dysjunction.

The “t” test revealed significant differences in the degree of mineralization of dental enamel values before and after the maxillary dysjunction appliance (p=0.0046). Variations in the standard deviation suggested that the sample became less homogeneous at the second study point. Minimum amplitude values remained close, whereas maximum amplitude values increased, which indicated dispersion of values towards the end of orthodontic therapy. The degree of mineralization values gathered at the end of the study ranged from 2.11 to 4.05, with a mean value of 3.08. Table 1 shows the descriptive analysis, and Chart 1 shows the sample behavior.

**Table 1:** Degree of enamel mineralization before and after maxillary dysjunction

	Before (n=20)	After (n=20)	“T” test
mean	2,7	5,78	0,046 *
SD	0,9	2	
minimum	1,33	1,92	
maximum	5,08	10,17

**Chart 2:**
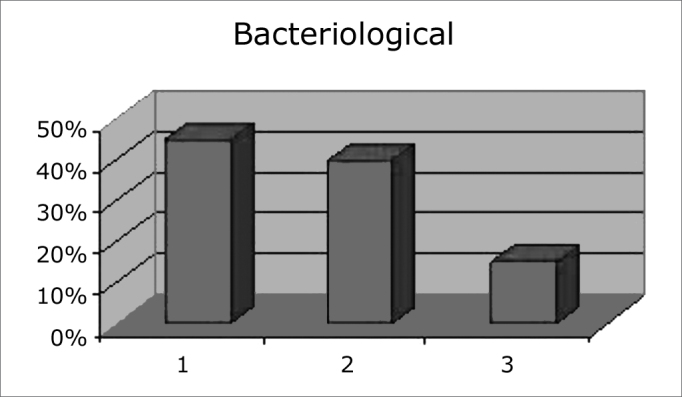
Potential of dental caries before and after the maxillary dysjunction appliance.

Salivary bacteriological qualitative measurement data were tabulated; the colorimetric test showed that the potential for dental caries decreased in 45% of the sample, it remained unaltered in 40% of the sample, and increased in only 15% of the sample - Chart 2.

## DISCUSSION

Placing orthodontic appliances in the mouth - such as a Hyrax-type dysjunction appliance - may affect the physical, chemical and biological features of the mouth and the bacterial plaque, which may increase caries and periodontal disease.^15^

The dysjunction appliance in mouth breathers increased significantly the degree of enamel demineralization in our sample; the mean value was 3.08. Based on the reference clinical parameters,^16^ values from 0 to 5 are within normal limits; these values, therefore, may be considered as “HEALTHY” in histology, not requiring any intervention. Well-adapted appliances and latest-generation cement with gradual fluoride release, and concerns with motivating patients to practice oral hygiene may have minimized possible decalcification.

Saliva diagnosis with No Caries 1 and 2 evaluated the pH and the microbiological aspects. The presence of microorganisms in saliva revealed its status at the collection time and vulnerability even when clinically no caries or gingivitis was seen. The principle of the No Caries test is based on an in vitro reaction by *Streptococcus Mutans* and *Neisseria Bucalis,* which alter the pH in the solution and change its color by hydrolyzing substrates.^17^, ^18^

The response to a cariogenic environment varies considerably among individuals; the presence of the maxillary dysjunction appliance increased the potential for caries in only 15% of the sample. These patients were probably less receptive to oral hygiene orientations, which were reiterated in every visit. The 45% that reduced the potential for caries, and the 40% in which this potential remained unchanged regardless of the appliance, possibly because of the knowledge that they would be tested, were induced to perform adequate self-care.

It should be noted that caries do not develop in the absence of dental plaques or fermentation of diet carbohydrates; it may be considered as a diet-bacterial disease.^19^ Several microorganisms in dental plaques may cause caries, such as *Streptococcus mutans, Streptococcus sobrinus,* some lactobacilli species, species of Actinomyces, *Streptococcus nonmutans*, and other fermenting bacteria. The caries-associated microbial virulence strongly associated with caries includes the ability to produce acids and to maintain a low pH, which results in dental demineralization. *Streptococcus mutans* has all these features, thus its etiological role in caries.^19^ The presence of a fixed dysjunction appliance makes hygiene more difficult, but not impossible. Therefore, frequent visits from members of a multidisciplinary team - Otorhinolaryngology (assessing upper airways) and Dentistry (hygiene and diet orientations, bacterial plaque prevention, well-adapted and cemented appliances) - were essential for the results of this study.

## CONCLUSION

1. There was a statistically significant difference in the degree of dental enamel mineralization in mouth breathers after using the maxillary dysjunction appliance; it remained, however, within the normal clinical range.
2. The potential for dental caries increased during orthodontic therapy in a few patients.
3. A multidisciplinary approach contributed to microbiologic dental caries control in mouth breathers.
